# Serum Nutritional Biomarkers and Their Associations with Sleep among US Adults in Recent National Surveys

**DOI:** 10.1371/journal.pone.0103490

**Published:** 2014-08-19

**Authors:** May A. Beydoun, Alyssa A. Gamaldo, Jose A. Canas, Hind A. Beydoun, Mauli T. Shah, Jessica M. McNeely, Alan B. Zonderman

**Affiliations:** 1 National Institute on Aging, NIA/NIH/IRP, Baltimore, Maryland, United States of America; 2 Pediatric Endocrinology, Diabetes and Metabolism Nemours Children's Clinic, Jacksonville, Florida, United States of America; 3 Graduate program in Public Health, Eastern Virginia Medical School, Norfolk, Virginia, United States of America; 4 Department of Psychology, University of Maryland, Baltimore County, Baltimore, Maryland, United States of America; University of Rochester, United States of America

## Abstract

**Background:**

The associations between nutritional biomarkers and measures of sleep quantity and quality remain unclear.

**Methods:**

Cross-sectional data from the National Health and Nutrition Examination Surveys (NHANES) 2005–2006 were used. We selected 2,459 adults aged 20–85, with complete data on key variables. Five sleep measures were constructed as primary outcomes: (**A**) Sleep duration; (**B**) Sleep disorder; (**C**) Three factors obtained from factor analysis of 15 items and labeled as “Poor sleep-related daytime dysfunction” (**Factor 1**), “Sleepiness” (**Factor 2**) and “Sleep disturbance” (**Factor 3**). Main exposures were serum concentrations of key nutrients, namely retinol, retinyl esters, carotenoids (*α*-carotene, *β*-carotene, *β*-cryptoxanthin, lutein+zeaxanthin, lycopene), folate, vitamin B-12, total homocysteine (tHcy), vitamin C, 25-hydroxyvitamin D (25(OH)D) and vitamin E. Main analyses consisted of multiple linear, logistic and multinomial logit models.

**Results:**

Among key findings, independent inverse associations were found between serum vitamin B-12 and sleep duration, 25(OH)D and sleepiness (as well as insomnia), and between folate and sleep disturbance. Serum total carotenoids concentration was linked to higher odds of short sleep duration (i.e. 5–6 h per night) compared to normal sleep duration (7–8 h per night).

**Conclusions:**

A few of the selected serum nutritional biomarkers were associated with sleep quantity and quality. Longitudinal studies are needed to ascertain temporality and assess putative causal relationships.

## Introduction

Serum nutritional biomarkers' role in health and disease has evolved from a marker of deficiency in one bodily system (e.g. vitamin D and bone), to non-classical metabolic roles, ultimately altering myriad of chronic conditions spanning the pulmonary, immune, digestive, muskuloskeletal, endocrine, cardiovascular, and central nervous systems (CNS). For CNS conditions, studies have linked cognitive and affective disorders (e.g. dementia, cognitive decline and depression) to variations in serum nutritional biomarkers, including carotenoids, retinol (or retinyl esters), folate, vitamin B-12, total homocysteine (tHcy), vitamin C, D and E (e.g. [1], [2], [3], [4], [5], [6], [7], [8], [9], [10], [11], [12], [13], [14]). Despite this trend, and the known association between sleep and cognitive as well as affective disorders [15], [16], [17], limited epidemiological research has explored how sleep quantity and quality may be related to serum nutritional biomarkers [18], [19], [20], [21], [22], [23], [24], [25], [26].

Among those biomarkers, carotenoids were inversely related to metabolic syndrome and depressive symptoms and positively related to cardiovascular health [4], [27], [28]. Of around 40 carotenoids found in plant roots, leaves, shoots, seeds, fruit, and flowers, only a handful are ubiquitous in human serum, the most common being α-carotene, β-carotene, lutein+zeaxanthin (often grouped together), β-cryptoxanthin and lycopene [28]. Serum carotenoid concentrations are considered both markers of antioxidant capacity [29] and of total fruit and vegetable intake [28]. While dietary and/or supplemental *β*-carotene is the main source of pro-vitamin A, serum retinol and retinyl esters are direct measures of vitamin A bioavailability. Folate, vitamin B-12 and tHcy are often grouped as 1-C metabolism micronutrients as both folate and vitamin B-12 are needed to reduce the level of tHcy through methylation reactions in the CNS [30]. tHcy is now known to increase the risk of cardiovascular disease and as well as cognitive decline and dementia [31], while serum folate was consistently related to reduced risk of depression.(e.g. [1], [2]) Other micronutrients with known antioxidant effects are vitamins C (main dietary source: citrus fruits) and E (main dietary sources: plant oils) [32]. Finally, serum 25(OH)D, main sources: sunlight and dairy products) was specifically shown to have protective effects against cardiovascular disease [33], [34], cancer [35], [36] and infection [37], [38].

Recent research has evaluated relationships between inadequate sleep and adiposity or obesity-related metabolic disorders [39], [40], [41], [42]. Restricting sleep in a controlled setting suggested sleep's link to weight gain/obesity is explained by increases in ghrelin (the hormone stimulating appetite) and decreases in leptin (the hormone signaling satiety) [43]. Such hormonal changes can affect an individual's appetite and eating patterns. Given that eating patterns are key determinants of serum nutritional biomarker concentrations (e.g. serum folate vs. dietary quality [1]), the direct association between those biomarkers and sleep should be evaluated. Several studies have observed an association between dietary intakes of macro and micronutrients and various measures of sleep such as sleep duration, sleep onset, number of awakenings, wake after sleep, sleep medication use, total napping, obstructive sleep apnea, insomnia [44], [45], [46], [47], [48], [49], [50], [51], [52]. A recent review also discusses the possible mechanisms by which diet may influence sleep [53]. Unlike our present study, much of the previous research, has typically used dietary assessment methods that are prone to inaccurate recall of individual dietary behavior or measurement error which, in turn, may be differential by outcome status and thus bias the final measure of association possibly away from the null.

In our present study, we aimed at examining the cross-sectional associations between several nutritional biomarkers and measures of sleep quantity and quality, controlling for dietary intake of specific nutrients and supplement use as well as other potentially confounding socio-demographic, lifestyle and health-related factors, using a nationally representative sample of US adults. Although this study is largely exploratory given the paucity of evidence thus far, we hypothesize that in general, nutritional biomarkers that are linked to lower oxidative stress, including carotenoids, retinol+retinyl esters, B-vitamins (folate and B-12), vitamin C, D and E, are linked to better sleep outcomes whereas tHcy is linked to poorer outcomes.

## Materials and Methods

### Database and study subjects

The National Health and Nutrition Examination Surveys (NHANES) was conducted according to the guidelines laid down in the Declaration of Helsinki, and all procedures involving human subjects/patients were approved by the Institutional Review Board of the National Center for Health Statistics, Centers for Disease Control. Written or verbal informed consent was obtained from all participants; verbal consent was witnessed and formally recorded [54].

NHANES consist of cross-sectional surveys providing nationally representative data on the health and nutritional status of the U.S. civilian population initiated in the early 1970s by the National Center for Health Statistics (NCHS), Centers for Disease Control (CDC), with waves of data being collected in non-continuous fashion. Since 1999, NHANES became a continuous survey. NHANES has a stratified, multistage probability cluster sampling design and includes an in-home interview for demographic and basic health information completed by trained staff and a health examination in a mobile examination center (MEC) completed by physicians, medical/health technicians, and dietary and health interviewers [54].

We selected adults aged 20–85 years from the NHANES 2005–2006 wave. Among the initial sample of 4,979 adults (2,387 men and 2,592 women) with complete basic demographic data (**Sample 1**), 3,464 had complete serum biomarker status. Among those in **Sample 1**, 2,980 participants had complete data on diet, physical activity, smoking status, supplement use, weight, height and serum cholesterol (**Sample 2**). Among those in **Sample 2**, complete data on sleep quality variables was available for 2,459 participants (**Sample 3**). **Sample 3** differed from **Sample 1** on some basic demographic variables: compared to those only in **Sample 1**, **Sample 3** participants were younger, non-Hispanic white, married, above 200% of the federal poverty line, and have greater than high school education level. This information allowed adjustment for selection bias beyond the oversampling of certain groups which was adjusted by using sampling weights (See statistical analysis section).

### Outcome assessment: Sleep quantity and quality measures

Sleep-related measures and scales were included in the 2005–06 NHANES as part of the Computer-Assisted Personal Interview system. The sleep questionnaire included items on sleep habits and disorders. Moreover, a subscale of eight questions, related to general productivity from the Functional Outcomes of Sleep Questionnaire [55], was also included (See **Method S1** of **File S1**). In total, we considered 28 questions for sleep indices. The first index was a measure of sleep duration (in hours), which was also categorized into “Very short” (<5 h per night), “Short” (5–6 h per night), “Normal” (7–8 h per night), and “Long” (≥9 h per night) sleep duration, based on previous studies (e.g. [44]). The second combined all reported sleep disorders, resulting in a binary composite variable (0 = no sleep disorder; 1 = at least one sleep disorder). Those disorders included “sleep apnea”, “insomnia”, “restless leg syndrome”, and “other sleep disorders”. An exploratory factor analytic approach was used on scale items that were measured on a 5-point Likert scale reflecting poorer sleep quality with the higher score. (See **Tables S1** and **S2** of **File S1**) Three factors were extracted, rotated using varimax orthogonal rotation and predicted using the regression method. Those were labeled as: **Factor 1**: “Poor sleep-related daytime dysfunction”; **Factor 2**: “Sleepiness” and **Factor 3**: “Sleep disturbance”. A confirmatory factor analysis was also conducted including those three factors by constraining factor loadings that were <0.40 in the exploratory stage to be zero and the error terms to be uncorrelated. The fit of the model is presented also in **Table S3** of **File S1** [56].

### Nutritional Biomarker assays

Using high performance liquid chromatography (HPLC) with photodiode array detection, serum concentrations of key biomarkers were measured. In this study, retinol and retinyl esters (defined as the sum of retinyl palmitate and retinyl stearate) were analyzed both separately and summed (vitamin A). Additionally, carotenoids such as α-carotene, β-carotene (cis+trans), β-cryptoxanthin, lutein+zeaxanthin, and lycopene were summed as total carotenoids (pro-vitamin A) and considered as exposures. Vitamin E was defined as the sum of α- and γ-tocopherol. Vitamin C was measured with HPLC with electrochemical detection [57], [58]. A similar grouping of serum antioxidant exposures was done elsewhere [4], [27].

Serum folate and vitamin B-12 were measured by Bio-Rad Laboratories “Quantaphase II Folate” radioassay kit [59], [60]. Serum tHcy was measured using “Abbott Homocysteine (HCY) assay”, a fully automated technique [61], [62]. Serum 25(OH)D was measured by radioimmunoassay (Diasorin Inc., Stillwater, Minnesota) [63]. Correlations between the key serum nutritional biormarkers are presented in **Table S4** of **File S1**.

### Covariates

Socio-demographic covariates included age, sex, race/ethnicity, education, marital status and family income, measured by the poverty-income ratio or the ratio of household income to the poverty line determined for that year multiplied by 100 (PIR<100% of the poverty line, 100%-<200%, ≥200%). Physical activity was measured using individual leisure-time activities that were elicited from participants in an open-ended manner. Those were coded for their intensity as assessed by metabolic equivalent (MET), which was then multiplied by duration of the activity and frequency (per week). This MET×hr/week value was summed for each participant with “none” considered sedentary (score = 0) [64], [65], [66] Cigarette smoking status was defined as “never”, “former” or “current smoker”. Previous studies found an inverse relationship between current smoking status and serum concentrations of folate and carotenoids among others [2], [27]. All NHANES participants were eligible for two 24-hr dietary recalls in the 2005–2006 wave. The first recall was administered during MEC exams, and the second 3–10 days later by telephone interview. To estimate nutrient intake in the diet, we utilized the average of the two 24-hr dietary recalls and a revised nutrient database that estimated nutrient composition per gram of USDA food code [67]. Total dietary intakes of alcohol, caffeine, fiber and selected antioxidants [total and individual carotenoids (µg/d), vitamin C and vitamin E (mg/d), folate (mcg/d), vitamin B-12 (mcg/d) and *n*-3 highly fatty acids (*n*-3 HUFA, sum of docosahexaenoice acid, eicospentaenoic acid, docosapentaenoic acid (C≥20), in g/d] were considered. Energy-adjusted associations between nutrients and outcome were obtained by entering total energy intake as a covariate. Using the MyPyramid Equivalents Database [68], cup equivalents of fruits and vegetables were estimated per individual, averaged over two 24-hr recalls. We excluded participants with only one 24-hr recall. Dietary supplement use over the past 30 days was categorized as follows: (0): non-users; (1): using one supplement; (2): using two supplements or more. Anti-depressant use was categorized with the Multum drug therapeutic category level 1 = 242 and level 2 = 249 [69], and coded as “having used an anti-depressant over the past 30 days” (1 = yes; 0 = no). Body mass index (BMI, kg.m^−2^) was computed using measured weight and height, while total cholesterol was measured with coupled reactions with cholesteryl ester hydrolase, cholesterol oxidase and peroxidase (Roche Hitachi Models 717 and 912). As a sensitivity analysis, total cholesterol was added as a covariate due to its high correlation with many of lipophillic micronutrients included in our analyses (e.g. vitamins A, D and E) [27].

Because co-morbid chronic conditions were associated with worse sleep [70], [71], [72], we adjusted for self-reported type 2 diabetes, cardiovascular disease (i.e., congestive heart failure, coronary heart disease, angina, heart attack or stroke) and cancer.

### Statistical analysis

Using Stata 11.0 [73], we first differences of continuous and categorical measures across sleep duration categories using *t*-test (from bivariate ordinary least square (OLS) regression models comparing means of continuous variables taking normal sleep duration as the common referent) and design-based F-test, respectively. Second, we conducted multiple OLS regression models with socio-demographic, lifestyle and dietary factors as predictors of serum nutritional biomarkers, to test associations between various covariates with the key exposures of interest. Third, we tested the main associations between serum nutritional biomarkers (standardized *z*-scores) and the five sleep quality measures, adjusting for all covariates (entered simultaneously), using multiple OLS and logistic regression models. Four different types of models were ran for each of the outcomes of interest, specifycing main exposure variables in different ways (e.g. combining carotenoids vs. not), and compared using R^2^ for OLS models and design-based F-test for logistic regression models. Finally, multinomial logit regression models with very short, short, and long sleep durations were compared to normal sleep duration in their association with serum nutritional biomarker levels. Moreover, we also applied multinomial logit models to obtain distinctive associations between various sleep disorders and serum nutritional biomarkers. Due to low disorder prevalence (<1%), only two categories were compared to the “none” category, namely 1 = insomnia only, 2 = all others, including sleep apnea, restless leg syndrome and multiple disorders.

In all analyses, to provide an unbiased estimate for the standard errors, given sampling design complexity, masked variance units were used to estimate variances utilizing the Taylor series linearization method. MEC exam weights were incorporated in the analysis to correct for unequal probability of sampling for certain population groups and obtain population estimates of means, proportions and regression coefficients [74]. This was done by using Stata survey commands and specifying weights, strata and primary sampling units (PSU) [73].

Given missing data particularly in sleep measures, we constructed a two-stage Heckman selection model [75], [76], as was done previously in another study using a similar sample [2], to account for potential selection bias in all main analyses.

All p-values presented are 2-tailed, with p<0.05 considered statistically significant and p<0.10 marginally significant, before correction for multiple testing. The latter was done using a familywise bonferroni procedure, with a family of hypotheses defined by the sleep outcome, assuming content independence [77]. A similar approach was adopted in a previous study with cognitive decline outcomes [78]. For the sleep measures, statistical significance criterion for p-values and p-values for trend were reduced to p = 0.05/5 = 0.010 (marginal significance: p = 0.10/5 = 0.020).

## Results

### Study sample characteristics

**Table 1** presents the distribution of sample characteristics by sleep duration categories.Generally, participants with very short or short duration of sleep were less likely to be non-Hispanic Whites compared to those with normal sleep duration (56.3% and 67.8% vs. 77.3%). They were also less likely to be married (52.8% and 60.5% vs. 67.3%%) and to have >HS education (46.8% and 58.6% vs. 65.1%) or ≥200% PIR (55.2% and 72.2% vs. 75.6%). Similarly, the prevalence of current smoking was significantly higher among very short and short sleepers compared to normal duration sleepers (50.0% and 26.2% vs. 18.3%). Very short sleep duration was also associated with lower mean physical activity compared to normal sleep (5.2 vs. 7.9). The prevalence of type 2 diabetes, cardiovascular disease and cancer was also the lowest among normal sleepers. Compared to normal sleepers (10.6%), short sleepers were less likely to use anti-depressant medication (8.3%) whereas long sleepers were more likely to do so (20.9%). As expected, Factors 1 and 3 as well as higher prevalence of sleep disorders were directly linked to shorter sleep duration. Short and/or very short sleep duration compared to normal duration was associated with lower serum levels of retinyl esters, total carotenoids (mainly α-carotene, β-carotene and β-cryptoxanthin), vitamins C, E, folate and 25(OH)D. A very short sleep duration was associated with a higher tHcy level.

**Table 1:** Selected baseline characteristics of NHANES 2005–06 participants by sleep duration categories (*n* = 2,459)^1^^,^ 2.

	Sleep duration categories			
	(n = 114)		(n = 775)		(n = 1307)		(n = 173)	
	Very short <5 h		Short 5–6 h		Normal 7–8 h		Long ≥9 h	
	Mean, %	(SE)	Mean, %	(SE)	Mean, %	(SE)	Mean, %	(SE)
**Socio-demographic, lifestyle and health-related factors**								
Age (y)	45.3	(1.6)	45.1	(0.9)	46.0	(0.9)	47.5	(2.1)
Race/ethnicity, %^4^								
Non-Hispanic White	56.3	(7.5)	67.8	(2.9)	77.3	(2.5)	70.8	(4.6)
Non-Hispanic black	25.3	(6.2)	14.8	(2.7)	6.3	(1.1)	11.4	(3.0)
Mexican-American	6.3	(1.8)	9.0	(1.1)	6.9	(1.0)	7.1	(1.6)
Other ethnicity	12.0	(4.0)	8.5	(1.5)	9.0	(1.4)	10.7	(2.4)
Married, %^4^	52.8	(4.9)	60.5	(2.5)	67.3	(1.8)	62.6	(4.9)
Education, %^4^								
<High School	4.6	(1.6)	5.3	(1.0)	4.6	(0.9)	7.2	(1.9)
High School	48.6	(4.7)	36.0	(3.4)	30.3	(2.5)	40.9	(3.8)
>High School	46.8	(4.6)	58.6	(3.5)	65.1	(2.6)	51.9	(4.1)
Poverty Income Ratio, %^4^								
<100%	16.4	(4.1)	9.9	(1.5)	7.1	(0.9)	14.0	(3.1)
100%-<200%	28.4	(6.0)	17.9	(2.0)	17.3	(1.4)	30.0	(4.4)
≥200%	55.2	(6.5)	72.2	(2.9)	75.6	(2.0)	59.0	(4.7)
Smoking status, %^4^								
Never smoker	35.6	(4.9)	48.4	(2.1)	55.3	(2.5)	56.5	(5.4)
Former smoker	14.4	(5.8)	25.5	(1.6)	26.4	(1.8)	19.5	(4.0)
Current smoker	50.0	(7.1)	26.2	(2.3)	18.3	(1.5)	24.0	(4.3)
Physical activity, *Mets.hr.wk^−1^*	5.2	(1.0)^4^	7.6	(0.7)	7.9	(0.5)	6.4	(1.1)
Body mass index, kg.m^−2^	30.2	(0.7)^4^	29.6	(0.3)^4^	27.9	(0.4)	28.5	(0.7)
Serum total cholesterol, *mg/dL*	204.8	(5.3)	199.4	(2.0)	200.2	(1.4)	200.6	(2.1)
History of selected chronic conditions								
Type 2 diabetes, %^3^	16.3	(4.5)	8.2	(1.0)	6.5	(0.8)	7.0	(3.1)
Cardiovascular disease, %^3^	12.3	(3.6)	7.4	(1.3)	5.5	(0.9)	12.7	(3.8)
Cancer, %^3^	9.9	(2.9)	4.9	(0.8)	7.8	(0.5)	11.4	(3.4)
Anti-depressant medication use, % yes^4^	10.6	(3.7)	8.3	(1.6)	10.6	(0.7)	20.9	(2.7)
**Sleep outcome variables**								
Factor 1: Poor sleep-related daytime dysfunction	+0.21	(0.19)	+0.09	(0.04)^4^	−0.07	(0.02)	+0.01	(0.11)
Factor 2: Sleepiness	+0.65	(0.11)^4^	+0.19	(0.05)^4^	−0.07	(0.02)	+0.09	(0.08)
Factor 3: Sleep disturbance	+0.71	(0.14)^4^	+0.17	(0.04)^4^	−0.13	(0.03)	−0.11	(0.08)
Sleep duration (h per night)	+3.67	(0.07)^4^	+5.74	(0.01)^4^	+7.45	(0.02)	+9.39	(0.07)^4^
Sleep disorders, %^4^	22.2	(6.3)	8.2	(1.2)	4.9	(0.8)	6.1	(3.5)
Insomnia only, %	13.7	(0.5)	4.8	(0.8)	3.9	(0.8)	5.5	(3.4)
Sleep apnea only, %	2.2	(1.7)	1.2	(0.5)	0.4	(0.2)	0.0	(0.0)
Restless leg syndrome only, %	0.8	(0.8)	0.4	(0.2)	0.0	(0.0)	0.0	(0.0)
All others and multiple, %	0.0	(0.0)	0.8	(0.4)	0.0	(0.0)	0.0	(0.0)
**Nutritional biomarkers**								
Retinol, µ*mol/L*	2.11	(0.07)	2.10	(0.03)	2.14	(0.02)	2.02	(0.08)
Serum retinyl esters, µ*mol/L*	0.10	(0.01)	0.11	(0.00)^4^	0.12	(0.00)	0.12	(0.01)
*Total retinol+retinyl esters*, µ*mol/L*	2.22	(0.08)	2.21	(0.03)	2.26	(0.02)	2.14	(0.08)
α-Carotene, µ*mol/L*	0.06	(0.01)^4^	0.08	(0.00)^4^	0.10	(0.01)	0.09	(0.01)
β-Carotene, µ*mol/L*	0.24	(0.02)^4^	0.35	(0.02)^4^	0.43	(0.02)	0.41	(0.06)
β-Cryptoxanthin, µ*mol/L*	0.16	(0.01)	0.17	(0.01)^4^	0.19	(0.01)	0.18	(0.01)
Lutein+zeaxanthin, µ*mol/L*	0.28	(0.01)	0.28	(0.01)	0.30	(0.01)	0.29	(0.02)
Lycopene, µ*mol/L*	0.45	(0.02)	0.47	(0.01)	0.45	(0.01)	0.46	(0.02)
*Total carotenoids*, µ*mol/L*	1.19	(0.05)^4^	1.35	(0.04)^4^	1.47	(0.03)	1.42	(0.10)
Vitamin E, µ*mol/L*	28.6	(1.4)	28.2	(0.7)^3^	30.2	(0.4)	29.2	(1.1)
Vitamin C, µ*mol/L*	47.7	(4.1)^3^	50.0	(1.4)^4^	56.7	(1.0)	57.6	(3.1)
Folate, *nmol/L*	25.7	(1.5)^4^	28.9	(1.1)^4^	32.6	(0.7)	34.4	(2.4)
Vitamin B-12, *pmol/L*	629.6	(231.5)	394.0	(11.5)	402.4	(9.2)	373.4	(16.0)
tHcy, µ*mol/L*	9.2	(0.3)^4^	8.4	(0.1)	8.1	(0.1)	7.8	(0.2)
25(OH)D, *ng/mL*	18.0	(0.8)^4^	21.4	(0.5)^3^	22.5	(0.4)	23.0	(1.1)
**Dietary Intakes**								
Total Energy Intake, *kcal/d*	2,182	(126)	2,375	(51)^3^	2,241	(38)	2,100	(83)^3^
Alcohol intake, *g/d*	10.0	(2.6)	11.2	(1.2)	10.3	(1.1)	8.2	(1.5)
Caffeine, mg/d	302.6	(39.6)^4^	191.2	(8.3)	177.9	(5.5)	155.7	(12.3)
Fiber, g/d	13.7	(1.0)^4^	15.9	(0.3)^3^	16.7	(0.4)	14.3	(0.7)^4^
α-carotene, µ*g/d*	250.0	(39.7)^4^	369.0	(38.8)^3^	464.0	(30.5)	402.1	(67.2)
β-carotene, µ*g/d*	1,378.1	(176.0)^4^	1,915.4	(103.8)^4^	2,437.1	(124.4)	1,871.7	(216.2)
β-Cryptoxanthin, µ*g/d*	135.4	(24.6)	125.8	(10.1)	133.6	(6.5)	114.5	(91.2)
Lutein+zeaxanthin, µ*g/d*	1,091.5	(140.6)^4^	1,336.3	(96.7)^3^	1,619.3	(95.0)	1,149.5	(130.4)^4^
Lycopene, µ*g/d*	5,008.8	(1,146)	5,569.7	(316.3)	5,864.8	(298.3)	4,777.7	(444.3)
*Total carotenoids*, µ*g/d*	8,992	(1,363)^3^	10,863	(460)^4^	12,492	(418)	9,785^4^	(753)
vitamin C, *mg/d*	86.7	(9.3)	84.2	(4.0)	92.3	(3.1)	76.5^3^	(4.8)
vitamin E, *mg/d*	6.6	(0.6)^3^	7.6	(0.2)	7.8	(0.2)	6.7	(0.2)^4^
*n*-3 HUFA, g/d	0.12	(0.02)	0.14	(0.02)	0.17	(0.01)	0.16	(0.03)
Folate, µ*g*/d	362.0	(21.3)^4^	413.0	(8.0)	434.7	(8.9)	386.4^4^	(10.2)
Vitamin B-12, µ*g*/d	4.7	(0.4)^4^	5.7	(0.3)	5.9	(0.2)	5.3	(0.2)^3^
Dietary supplement use, past 30 d								
None	60.8	(5.1)	41.0	(2.2)	35.9	(1.9)	30.5	(3.0)
One	16.5	(4.6)	29.3	(2.0)	28.1	(1.8)	33.8	(4.1)
Two or more	22.6	(3.8)	29.7	(2.2)	36.0	(1.7)	35.7	(4.3)
Fruits and vegetable intake, cup equivalent/d	2.31	(0.20)	2.54	(0.10)^3^	2.72	(0.08)	2.23^3^	(0.15)

*Abbreviations:* 25(OH)D = 25-hydroxyvitamin D; *n*-3 HUFA = *n*-3 highly unsaturated fatty acids; NHANES = National Health and Nutrition Examination Survey; SE = Standard error; tHcy = Total homocysteine.

1 Values are mean±SE or percent±SE. Sampling design complexity is taken into account in all analyses. This analysis is done among participants with complete data for sleep quality variables and other key variables of interest (n = 2,459).

2 P-value was based on design-based *F*-test if row variable is categorical and *t*-test if row variable is continuous (comparing normal sleep to each of the other categories).

3 P<0.05.

4 P<0.01 for null hypothesis of no difference by sleep duration category.

Similar to our findings with serum total carotenoids, short and very short sleep duration compared to normal was associated with lower intake of dietary total carotenoids. These associations were noted for α-carotene, β-carotene and lutein+zeaxanthin. Moreover, shorter sleep duration (vs. normal) was associated with higher total energy intake, higher caffeine, but lower vitamin E, folate, vitamin B-12 and fruits+vegetables intakes.

On the other hand, lower intakes of a number of nutrients and food groups were linked to longer sleep duration vs. normal and those included fiber, lutein+zeaxanthin, total carotenoids, vitamin E, vitamin B-12, fruits and vegetables as well as total energy intake.

### Socio-demographic, lifestyle and dietary predictors of nutritional biomarkers

**Tables 2** and **3** shows independent associations among socio-demographic, lifestyle and dietary factors and nutritional biomarkers. Generally, most serum biomarker levels were positively associated with age, except for 25(OH)D. Men had higher retinol and tHcy levels whereas the reverse was true for vitamin C, total carotenoids, folate and 25(OH)D. Additionally, Non-Hispanic Whites had consistently higher retinol, folate and 25(OH)D levels but lower total carotenoids, compared to Non-Hispanic blacks and Mexican-Americans. Other racial/ethnic differences were found for vitamin E and tHcy. Being married was linked to lower tHcy but higher vitamin E, total carotenoids and 25(OH)D levels. Former/current smoker status was related to lower vitamin C, total carotenoid, and folate status. PA was associated with higher total carotenoids and 25(OH)D levels, whereas BMI was inversely related to multiple biomarkers (i.e. retinol, retinyl esters, vitamin C, total carotenoids and 25(OH)D). Moreover, a history of type 2 diabetes was associated with higher serum retinol concentration, whereas a history of cardiovascular disease was related to lower retinyl esters, vitamin E and total carotenoids levels, and a history of cancer was linked to higher vitamin E status. Anti-depressant medication use was associated with a higher serum retinol concentration. Supplement use was positively associated with most nutritional biomarkers except for vitamin B-12 and 25(OH)D, with an inverse relationship found for tHcy. Higher energy intake was associated with lower folate and carotenoid status, while higher alcohol intake was linked to higher retinol, vitamin E, tHcy and 25(OH)D levels. Caffeine intake was inversely related to serum folate concentration but positively associated with tHcy serum level. Moreover, fiber intake was inversely related to retinol but positively associated with total carotenoid status. *β*-carotene and lycopene were both reflected in serum, while a higher lycopene intake was positively linked to retinyl ester but inversely related to tHcy. Higher intakes of folate, vitamin B-12, vitamins C and E were marked by their higher respective serum levels. Moreover, vitamin E intake was positively while folate intake was inversely associated with tHcy. Dietary *n*-3 HUFA was linked to higher serum total carotenoid status. When adjusting for serum cholesterol in the models (data not shown), results were not altered.

**Table 2:** Socio-demographic, lifestyle and dietary predictors of nutritional biomarkers (retinol, retinyl esters, vitamin C, vitamin E and total carotenoids): OLS multiple regression analyses; NHANES 2005–06 (*n* = 2,459).

	Retinol µ*mol/L*			Retinyl esters µ*mol/L*			Vitamin C µ*mol/L*			Vitamin E µ*mol/L*			Total carotenoids µ*mol/L*		
	β	(SEE)	P-value	β	(SEE)	P-value	β	(SEE)	P-value	β	(SEE)	P-value	β	(SEE)	P-value
**Socio-demographic, lifestyle factors**															
Age (y)	**+0.006**	**(0.001)**	**<0.001**	+0.000	(0.000)	0.061	**+0.115**	**(0.041)**	**0.014**	**+0.204**	**(0.011)**	**<0.001**	**+0.004**	**(0.001)**	**0.001**
Female vs. male	**−0.226**	**(0.046)**	**<0.001**	−0.005	(0.004)	0.20	**+7.674**	**(2.100)**	**0.002**	+0.161	(0.506)	0.76	**+0.153**	**(0.034)**	**<0.001**
Race/ethnicity															
Non-Hispanic White	Ref			Ref			Ref			Ref			Ref		
Non-Hispanic black	**−0.244**	**(0.036)**	**<0.001**	+0.003	(0.004)	0.50	+2.617	(1.633)	0.13	**−2.113**	**(0.510)**	**0.001**	**+0.176**	**(0.054)**	**0.005**
Mexican-American	**−0.168**	**(0.029)**	**<0.001**	−0.003	(0.005)	0.57	+0.476	(2.276)	0.84	+1.251	(0.819)	0.147	**+0.175**	**(0.061)**	**0.012**
Other ethnicity	**−0.186**	**(0.031)**	**<0.001**	**−0.011**	**(0.004)**	**0.013**	+1.759	(2.814)	0.54	−0.610	(1.019)	0.558	+0.117	(0.061)	0.073
Married vs. non-married	−0.037	(0.030)	0.24	−0.001	(0.003)	0.77	+0.671	(1.345)	0.63	**+1.520**	**(0.587)**	**0.020**	**+0.114**	**(0.037)**	**0.007**
Education															
<High School	Ref			Ref			Ref			Ref			Ref		
High School	+0.032	(0.047)	0.50	+0.005	(0.008)	0.60	−2.029	(2.527)	0.44	−0.649	(0.995)	0.70	−0.020	(0.074)	0.79
>High School	+0.018	(0.059)	0.77	+0.011	(0.007)	0.15	−1.351	(2.631)	0.62	−0.362	(0.910)	0.52	−0.095	(0.083)	0.28
Poverty Income Ratio															
<100%	Ref			Ref			Ref			Ref			Ref		
100%-<200%	+0.027	(0.055)	0.63	+0.000	(0.005)	0.99	−1.170	(1.460)	0.44	−0.431	(0.968)	0.66	+0.006	(0.072)	0.69
≥200%	+0.013	(0.052)	0.81	−0.004	(0.005)	0.42	+1.490	(1.392)	0.30	+0.714	(0.966)	0.47	−0.021	(0.051)	0.69
Smoking status															
Never smoker	Ref			Ref			Ref			Ref			Ref		
Former smoker	+0.022	(0.019)	0.28	+0.002	(0.006)	0.78	−0.921	(1.948)	0.64	−0.928	(0.669)	0.19	−0.055	(0.064)	0.69
Current smoker	−0.020	(0.033)	0.56	−0.007	(0.004)	0.12	**−9.414**	**(1.491)**	**<0.001**	−0.877	(0.612)	0.17	**−0.257**	**(0.052)**	**<0.001**
Physical activity, *Mets.hr.wk^−1^*	+0.001	(0.001)	0.17	+0.000	(0.000)	0.90	+0.032	(0.032)	0.33	−0.015	(0.010)	0.14	**+0.003**	**(0.001)**	**0.009**
Body mass index, kg.m^−2^	**−0.006**	**(0.002)**	**0.003**	**−0.001**	**(0.000)**	**0.001**	**−0.773**	**(0.064)**	**<0.001**	+0.022	(0.024)	0.37	**−0.028**	**(0.002)**	**<0.001**
History of selected chronic conditions															
Type 2 diabetes	**+0.156**	**(0.042)**	**0.002**	+0.006	(0.006)	0.38	−3.988	(1.930)	0.057	+1.494	(1.215)	0.24	−0.115	(0.064)	0.09
Cardiovascular disease	0.048	(0.111)	0.674	**−0.016**	**(0.006)**	**0.024**	−0.029	(2.627)	0.991	**−3.311**	**(0.973)**	**0.004**	**−0.266**	**(0.056)**	**<0.001**
Cancer	+0.124	(0.064)	0.071	+0.007	(0.008)	0.41	+2.501	(2.886)	0.400	**+2.031**	**(0.714)**	**0.012**	+0.040	(0.056)	0.48
Anti-depressant medication use	**+0.127**	**(0.042)**	**0.009**	−0.010	(0.005)	0.080	−1.074	(2.329)	0.65	+0.730	(1.079)	0.51	−0.099	(0.062)	0.13
**Dietary Intakes**															
Total Energy Intake, *kcal/d*	+0.000	(0.000)	0.12	−0.000	(0.000)	0.10	−0.001	(0.064)	0.26	−0.000	(0.000)	0.10	**−0.000**	**(0.000)**	**0.001**
Alcohol intake, *g/d*	**+0.003**	**(0.001)**	**0.001**	+0.000	(0.000)	0.13	+0.045	(0.035)	0.22	**+0.040**	**(0.012)**	**0.007**	+0.001	(0.001)	0.19
Caffeine, mg/d	−0.000	(0.000)	0.30	−0.000	(0.000)	0.36	−0.004	(0.003)	0.26	−0.001	(0.001)	0.42	+0.000	(0.000)	0.46
Fiber, g/d	**−0.005**	**(0.002)**	**0.032**	+0.000	(0.000)	0.85	+0.269	(0.130)	0.06	−0.003	(0.049)	0.94	**+0.007**	**(0.003)**	**0.022**
α-carotene, µ*g/d*	+0.000	(0.000)	0.62	+0.000	(0.000)	0.16	−0.001	(0.001)	0.52	−0.001	(0.001)	0.26	+0.000	(0.000)	0.07
β-carotene, µ*g/d*	−0.000	(0.000)	0.37	+0.000	(0.000)	0.45	+0.000	(0.000)	0.45	+0.000	(0.000)	0.43	**+0.000**	**(0.000)**	**0.013**
β-Cryptoxanthin, µ*g/d*	−0.000	(0.000)	0.80	−0.000	(0.000)	0.38	+0.000	(0.004)	0.94	−0.000	(0.002)	0.93	+0.000	(0.000)	0.12
Lutein+zeaxanthin, µ*g/d*	+0.000	(0.000)	0.45	+0.000	(0.000)	0.77	−0.000	(0.000)	0.40	−0.000	(0.000)	0.65	+0.000	(0.000)	0.14
Lycopene, µ*g/d*	+0.000	(0.000)	0.74	**+0.000**	**(0.000)**	**0.009**	−0.000	(0.011)	0.97	−0.000	(0.000)	0.36	**+0.000**	**(0.000)**	**0.022**
vitamin C, *mg/d*	+0.000	(0.000)	0.34	+0.000	(0.000)	0.60	**+0.070**	**(0.011)**	**<0.001**	+0.005	(0.004)	0.23	+0.000	(0.000)	0.50
vitamin E, *mg/d*	+0.002	(0.006)	0.68	+0.001	(0.001)	0.13	+0.014	(0.103)	0.89	**+0.182**	**(0.053)**	**0.004**	+0.002	(0.005)	0.67
*n*-3 HUFA, g/d	+0.024	(0.044)	0.59	−0.008	(0.007)	0.30	+1.684	(1.767)	0.36	+0.805	(1.159)	0.50	**+0.221**	**(0.069)**	**0.006**
Folate, µ*g*/d	−0.000	(0.017)	0.98	+0.000	(0.000)	0.061	−0.004	(0.003)	0.25	**+0.003**	**(0.002)**	**0.037**	−0.000	(0.000)	0.95
Vitamin B-12, µ*g*/d	+0.006	(0.004)	0.10	**+0.010**	**(0.003)**	**0.032**	**+0.274**	**(0.122)**	**0.041**	−0.016	(0.050)	0.75	−0.005	(0.004)	0.19
Dietary supplement use, past 30 d															
None	Ref			Ref			Ref			Ref			Ref		
One	**+0.076**	**(0.037)**	**0.044**	**+0.010**	**(0.004)**	**0.032**	**+5.736**	**(1.347)**	**0.001**	**+2.803**	**(0.716)**	**0.001**	+0.064	(0.035)	0.09
Two or more	**+0.226**	**(0.030)**	**<0.001**	**+0.030**	**(0.003)**	**<0.001**	**+17.198**	**(1.480)**	**<0.001**	**+8.419**	**(0.778)**	**<0.001**	**+0.266**	**(0.036)**	**<0.001**
Fruits and vegetable intake, cup equivalent/d	+0.000	(0.017)	0.98	−0.001	(0.002)	0.68	+0.661	(0.833)	0.44	−0.153	(0.252)	0.55	+0.033	(0.022)	0.16

**Table 3:** Socio-demographic, lifestyle and dietary predictors of nutritional biomarkers (folate, vitamin B-12, tHcy and 25(OH)D): OLS multiple regression analyses; NHANES 2005–06 (*n* = 2,459).

	Folate, *nmol/L*			Vitamin B-12, *pmol/L*			tHcy, µ*mol/L*			25(OH)D, *ng/mL*		
	β	(SEE)	P-value	β	(SEE)	P-value	β	(SEE)	P-value	β	(SEE)	P-value
**Socio-demographic, lifestyle factors**												
Age (y)	**+0.21**	**(0.03)**	**<0.001**	**+1.05**	**(0.29)**	**0.003**	**+0.069**	**(0.003)**	**<0.001**	**−0.059**	**(0.017)**	**0.004**
Female vs. male	**+4.23**	**(1.12)**	**0.002**	+38.44	(27.89)	0.19	**−1.471**	**(0.144)**	**<0.001**	**+1.164**	**(0.521)**	**0.041**
Race/ethnicity												
Non-Hispanic White	Ref			Ref			Ref					
Non-Hispanic black	**−4.94**	**(0.86)**	**<0.001**	+39.22	(27.95)	0.18	+0.461	(0.280)	0.120	**−8.363**	**(0.726)**	**<0.001**
Mexican-American	**−3.38**	**(1.36)**	**0.025**	+168.13	(96.25)	0.10	**−0.596**	**(0.160)**	**0.002**	**−5.212**	**(1.091)**	**<0.001**
Other ethnicity	**−3.33**	**(1.21)**	**0.014**	−22.83	(29.32)	0.45	−0.043	(0.289)	0.88	**−4.979**	**(0.842)**	**<0.001**
Married vs. non-married	+1.42	(1.05)	0.19	−12.71	(39.60)	0.75	**−0.252**	**(0.071)**	**0.003**	**+1.104**	**(0.842)**	**0.007**
Education												
<High School	Ref			Ref			Ref					
High School	−0.49	(1.70)	0.78	−228.4	(153.5)	0.16	+0.367	(0.320)	0.27	−0.984	(1.054)	0.37
>High School	+0.42	(1.55)	0.79	−219.1	(142.1)	0.14	+0.164	(0.323)	0.35	−1.256	(0.905)	0.19
Poverty Income Ratio												
<100%	Ref			Ref			Ref			Ref		
100%-<200%	−0.84	(1.01)	0.42	+32.20	(87.55)	0.72	−0.383	(0.400)	0.35	+0.772	(0.823)	0.36
≥200%	+0.33	(0.96)	0.73	−1.03	(67.59)	0.99	−0.515	(0.407)	0.23	+0.174	(0.854)	0.84
Smoking status												
Never smoker	Ref			Ref			Ref			Ref		
Former smoker	−1.73	(0.93)	0.082	−57.44	(24.1)	0.031	+0.065	(0.152)	0.68	−0.221	(0.414)	0.60
Current smoker	**−2.82**	**(0.83)**	**0.004**	−44.67	(26.36)	0.11	+0.468	(0.246)	0.08	−0.437	(0.505)	0.40
Physical activity, *Mets.hr.wk^−1^*	−0.010	(0.013)	0.52	−0.164	(0.492)	0.74	−0.007	(0.005)	0.15	**+0.027**	**(0.010)**	**0.020**
Body mass index, kg.m^−2^	−0.05	(0.11)	0.64	−2.969	(1.551)	0.075	−0.014	(0.010)	0.19	**−0.264**	**(0.029)**	**<0.001**
History of selected chronic conditions												
Type 2 diabetes	+0.02	(1.12)	0.99	+193.64	(161.91)	0.25	−0.013	(0.229)	0.95	−0.864	(0.680)	0.22
Cardiovascular disease	+3.03	(2.00)	0.15	−59.19	(53.65)	0.29	+0.628	(0.302)	0.06	−1.348	(0.9810	0.19
Cancer	−0.52	(1.66)	0.76	+105.79	(140.68)	0.46	+0.189	(0.175)	0.30	+0.706	(0.648)	0.29
Anti-depressant medication use	−0.64	(0.98)	0.53	−6.37	(39.19)	0.87	−0.252	(0.313)	0.08	+0.763	(0.436)	0.10
**Dietary Intakes**												
Total Energy Intake, *kcal/d*	**−0.001**	**(0.000)**	**0.015**	−0.005	(0.011)	0.63	−0.000	(0.000)	0.98	+0.000	(0.000)	0.82
Alcohol intake, *g/d*	−0.010	(0.014)	0.50	+0.509	(0.499)	0.32	**+0.012**	**(0.005)**	**0.037**	**+0.033**	**(0.012)**	**0.015**
Caffeine, mg/d	**−0.010**	**(0.002)**	**0.002**	−0.039	(0.035)	0.28	**+0.001**	**(0.000)**	**0.020**	+0.001	(0.001)	0.30
Fiber, g/d	−0.047	(0.079)	0.57	+1.206	(1.923)	0.54	−0.018	(0.010)	0.09	−0.005	(0.030)	0.87
α-carotene, µ*g/d*	−0.000	(0.001)	0.66	+0.000	(0.003)	0.97	−0.000	(0.000)	0.40	+0.000	(0.000)	0.89
β-carotene, µ*g/d*	−0.000	(0.000)	0.58	−0.002	(0.006)	0.79	−0.000	(0.000)	0.69	−0.000	(0.000)	0.92
β-Cryptoxanthin, µ*g/d*	−0.002	(0.003)	0.57	+0.014	(0.038)	0.72	−0.000	(0.000)	0.59	−0.000	(0.001)	0.84
Lutein+zeaxanthin, µ*g/d*	−0.000	(0.000)	0.35	+0.000	(0.003)	0.96	+0.000	(0.000)	0.38	+0.000	(0.000)	0.45
Lycopene, µ*g/d*	−0.000	(0.000)	0.18	−0.000	(0.001)	0.95	**−0.000**	**(0.000)**	**0.016**	−0.000	(0.000)	0.45
vitamin C, *mg/d*	+0.009	(0.008)	0.29	+0.108	(0.140)	0.45	−0.000	(0.001)	0.66	−0.002	(0.003)	0.95
vitamin E, *mg/d*	−0.125	(0.107)	0.26	−0.291	(2.063)	0.89	**+0.026**	**(0.010)**	**0.029**	+0.003	(0.06)	0.95
*n*-3 HUFA, g/d	+0.690	(1.354)	0.62	+18.026	(33.896)	0.60	−0.328	(0.235)	0.18	−1.007	(0.596)	0.11
Folate, µ*g*/d	**+0.017**	**(0.002)**	**<0.001**	+0.080	(0.050)	0.13	**−0.002**	**(0.000)**	**0.002**	+0.001	(0.001)	0.20
Vitamin B-12, µ*g*/d	−0.025	(0.049)	0.61	+0.502	(1.400)	0.73	+0.003	(0.013)	0.79	**+0.126**	**(0.027)**	**<0.001**
Dietary supplement use, past 30 d												
None	Ref			Ref			Ref			Ref		
One	**+5.01**	**(1.20)**	**0.001**	**+45.32**	**(26.78)**	**0.009**	**−0.407**	**(0.165)**	**0.027**	**+1.542**	**(0.343)**	**<0.001**
Two or more	**+11.98**	**(1.42)**	**<0.001**	+90.42	(30.34)	0.25	**−0.792**	**(0.159)**	**<0.001**	+2.666	(0.446)	0.22
Fruits and vegetable intake, cup equivalent/d	+0.48	(0.39)	0.23	−0.54	(8.31)	0.95	+0.040	(0.067)	0.56	0.327	(0.202)	0.13

*Abbreviations:* 25(OH)D = 25-hydroxyvitamin D; *n*-3 HUFA = *n*-3 highly unsaturated fatty acids; NHANES = National Health and Nutrition Examination Survey; SEE = Standard error of the estimate; tHcy = Total homocysteine.

### Nutritional biomarkers' association with sleep quantity and quality

Nutritional biomarkers' independent associations with sleep quantity and quality are shown in **Table 4**. In **Model 1**, serum nutritional biomarkers were entered separately as predictors along with other covariates. After familywise Bonferroni correction (type I error reduced to 0.01), a significant inverse association was found between sleep disorders and 25(OH)D level (per SD = 8.7 ng/mL: OR = 0.72; 95% CI: 0.58–0.91, p = 0.008). A shorter sleep duration was associated with a higher serum level of vitamin B-12 (per SD = 1154.4 pmol/L: β = −0.07±0.02, P = 0.002). Examining the three factors of sleep quality, inverse associations of Factor 1 (poor sleep-related daytime dysfunction) with retinyl esters, Factor 2 (Sleepiness) with 25(OH)D, and Factor 3 (Sleep disturbance) with three serum nutritional biomarkers (lutein+zeaxanthin, vitamin C and folate) were observed.

**Table 4:** Associations between serum nutritional biomarkers (per 1 SD increase) and sleep quality measures: Multiple OLS and logistic regression models, controlling for dietary intakes or supplement use: 2-stage Heckman selection models (N = 2,346); NHANES 2005–06^1^.

	Sleep duration		Sleep disorders			Factor 1: Poor sleep-related daytime dysfunction		Factor 2: Sleepiness		Factor 3: Sleep disturbance	
	β ±SEE	p-value	OR	(95% CI)	p- value	β±SEE	p-value	β±SEE	p- value	β±SEE	p- value
**MODEL 1** 2											
Retinol, per 0.63 µ*mol/L*	−0.05±0.03	0.19	0.78	(0.60;1.03)	0.08	−0.01±0.02	0.58	+0.01±0.02	0.69	+0.03±0.02	0.13
	R^2^ = 0.0753		**F(9,7) = 4.05, p = 0.039**			R^2^ = 0.0714		R^2^ = 0.0943		R^2^ = 0.0586	
Retinyl esters, per 0.08 µ*mol/L*	+0.03±0.02	0.24	0.80	(0.55;1.17)	0.23	**−0.05±0.02**	**0.007**	−0.02±0.02	0.37	+0.01±0.01	0.50
	R^2^ = 0.0748		F(9,7) = 1.99, p = 0.19			R^2^ = 0.0739		R^2^ = 0.0945		R^2^ = 0.0578	
α-Carotene, per 0.09 µ*mol/L*	−0.01±0.02	0.64	0.82	(0.52;1.31)	0.38	**−0.03±0.01**	**0.019**	−0.02±0.02	0.34	+0.01±0.02	0.76
	R^2^ = 0.074		F(9,7) = 0.39, p = 0.91			R^2^ = 0.0724		R^2^ = 0.0948		R^2^ = 0.0578	
β-Carotene, per 0.47 µ*mol/L*	−0.02±0.03	0.54	0.70	(0.43;1.15)	0.15	−0.04±0.02	0.07	**+0.06±0.03**	**0.047**	−0.04±0.02	0.07
	R^2^ = 0.0745		F(9,7) = 1.18, p = 0.42			R^2^ = 0.0727		R^2^ = 0.0979		R^2^ = 0.0592	
β-Cryptoxanthin, per 0.18 µ*mol/L*	+0.01±0.04	0.78	0.91	(0.51;1.61)	0.73	−0.00±0.02	0.99	**+0.06±0.03**	**0.015**	**−0.09±0.03**	**0.017**
	R^2^ = 0.0744		F(9,7) = 1.56, p = 0.28			R^2^ = 0.0713		R^2^ = 0.0973		R^2^ = 0.0624	
Lutein+zeaxanthin, per 0.17 µ*mol/L*	−0.02±0.03	0.60	**0.69**	**(0.51;0.94)**	**0.022**	+0.02±0.01	0.26	+0.00±0.02	0.91	**−0.05±0.01**	**0.007**
	R^2^ = 0.0745		F(9,7) = 1.44, p = 0.32			R^2^ = 0.0716		R^2^ = 0.0942		R^2^ = 0.0601	
Lycopene, per 0.21 µ*mol/L*	−0.02±0.04	0.61	0.87	(0.67;1.13)	0.27	−0.01±0.02	0.50	+0.03±0.02	0.07	+0.00±0.02	0.81
	R^2^ = 0.0746		F(9,7) = 2.71, p = 0.10			R^2^ = 0.0715		R^2^ = 0.0953		R^2^ = 0.0577	
Vitamin E, per 12.2 µ*mol/L*	−0.00±0.03	0.92	0.81	(0.61;1.09)	0.15	**−0.04±0.02**	**0.034**	−0.03±0.03	0.25	+0.01±0.02	0.78
	R^2^ = 0.0744		**F(9,7) = 4.26, p = 0.034**			R^2^ = 0.0730		R^2^ = 0.0949		R^2^ = 0.0578	
Vitamin C, per 26.6 µ*mol/L*	+0.04±0.05	0.42	**0.80**	**(0.65;0.98)**	**0.037**	−0.00±0.03	0.97	−0.04±0.03	0.18	**−0.05±0.02**	**0.006**
	R^2^ = 0.0751		F(9,7) = 0.72, p = 0.68			R^2^ = 0.0713		R^2^ = 0.0956		R^2^ = 0.0603	
Folate, per 20.3 *nmol/L*	+0.05±0.02	0.07	0.86	(0.60;1.22)	0.36	−0.03±0.02	0.06	**−0.04±0.02**	**0.028**	**−0.07±0.03**	**0.005**
	R^2^ = 0.0753		F(9,7) = 1.36, p = 0.35			R^2^ = 0.0721		R^2^ = 0.0958		R^2^ = 0.0622	
Vitamin B-12, per 1154.4 *pmol/L*	**−0.07±0.02**	**0.002**	0.93	(0.36;2.49	0.88	−0.01±0.01	0.53	+0.02±0.01	0.09	+0.03±0.01	0.08
	R^2^ = 0.0758		F(9,7) = 1.62, p = 0.27			R^2^ = 0.0713		R^2^ = 0.0945		R^2^ = 0.0581	
tHcy, per 4.9 µ*mol/L*	**−0.09±0.03**	**0.011**	0.87	(0.52;1.48)	0.60	−0.02±0.03	0.38	+0.02±0.03	0.42	+0.02±0.02	0.36
	R^2^ = 0.0764		**F(9,7) = 5.63, p = 0.016**			R^2^ = 0.0717		R^2^ = 0.0945		R^2^ = 0.0581	
25(OH)D, per 8.7 *ng/mL*	+0.07±0.04	0.10	**0.72**	**(0.58;0.91)**	**0.008**	+0.01±0.02	0.72	**−0.08±0.03**	**0.004**	−0.02±0.03	0.43
	R^2^ = 0.0766		F(9,7) = 2.02, p = 0.18			R^2^ = 0.0714		R^2^ = 0.1010		R^2^ = 0.0581	
**MODEL 2** 3	R^2^ = 0.0842		F(9,7) = 2.72, p = 0.10			R^2^ = 0.0785		R^2^ = 0.1193		R^2^ = 0.0744	
Retinol, per 0.63 µ*mol/L*	−0.06±0.04	0.12	0.89	(0.69;1.16)	0.37	+0.00±0.02	0.94	+0.04±0.02	0.10	**+0.03±0.02**	**0.036**
Retinyl esters, per 0.08 µ*mol/L*	+0.05±0.03	0.14	0.94	(0.66;1.35)	0.74	−0.03±0.02	0.09	−0.01±0.02	0.72	+0.02±0.03	0.41
α-Carotene, per 0.09 µ*mol/L*	−0.01±0.03	0.81	1.04	(0.75;1.46)	0.79	−0.02±0.02	0.20	**−0.09±0.02**	**0.002**	+0.05±0.02	0.05
β-Carotene, per 0.47 µ*mol/L*	−0.03±0.05	0.48	0.78	(0.51;1.21)	0.25	−0.03±0.02	0.17	**+0.09±0.03**	**0.003**	−0.03±0.02	0.17
β-Cryptoxanthin, per 0.18 µ*mol/L*	+0.03±0.05	0.56	1.25	(0.71;2.19)	0.41	+0.01±0.02	0.62	**+0.06±0.03**	**0.027**	−0.08±0.04	0.07
Lutein+zeaxanthin, per 0.17 µ*mol/L*	−0.01±0.04	0.72	0.74	(0.53;1.05)	0.09	**+0.04±0.01**	**0.005**	−0.02±0.02	0.45	**−0.05±0.02**	**0.034**
Lycopene, per 0.21 µ*mol/L*	−0.03±0.05	0.55	0.98	(0.74;1.30)	0.90	+0.00±0.02	0.91	+0.03±0.02	0.12	+0.01±0.02	0.48
Vitamin E, per 12.2 µ*mol/L*	−0.00±0.03	0.89	0.96	(0.70;1.30)	0.76	−0.04±0.03	0.12	−0.05±0.02	0.05	+0.03±0.03	0.38
Vitamin C, per 26.6 µ*mol/L*	+0.03±0.05	0.62	0.87	(0.69;1.10)	0.22	+0.01±0.03	0.71	−0.03±0.03	0.25	−0.02±0.01	0.19
Folate, per 20.3 *nmol/L*	+0.03±0.03	0.19	0.96	(0.71;1.28)	0.74	−0.02±0.01	0.21	−0.02±0.02	0.36	**−0.07±0.02**	**0.003**
Vitamin B-12, per 1154.4 *pmol/L*	**−0.08±0.03**	**0.006**	1.07	(0.56;2.06)	0.82	−0.00±0.01	0.75	**+0.03±0.01**	**0.043**	**+0.03±0.01**	**0.029**
tHcy, per 4.9 µ*mol/L*	**−0.07±0.03**	**0.015**	0.80	(0.47;1.38)	0.41	−0.03±0.03	0.28	+0.01±0.02	0.62	+0.01±0.03	0.78
25(OH)D, per 8.7 *ng/mL*	+0.08±0.04	0.10	**0.77**	**(0.62;0.96)**	**0.022**	+0.01±0.02	0.47	**−0.09±0.03**	**0.005**	−0.02±0.02	0.52
**MODEL 3** 4											
Total retinol+retinyl esters, per 0.64 µ*mol/L*	−0.04±0.03	0.24	0.77	(0.58;1.03)	0.08	−0.02±0.02	0.38	+0.01±0.02	0.78	+0.03±0.02	0.11
	R^2^ = 0.0751		F(9,7) = 2.86, p = 0.09			R^2^ = 0.0716		R^2^ = 0.0942		R^2^ = 0.0587	
Total carotenoids, per 0.82 µ*mol/L*	−0.02±0.03	0.45	0.70	(0.48;1.03)	0.07	−0.03±0.02	0.18	+0.05±0.03	0.07	**−0.04±0.02**	**0.032**
	R^2^ = 0.0746		F(9,7) = 0.91, p = 0.56			R^2^ = 0.0721		R^2^ = 0.0970		R^2^ = 0.0596	
**MODEL 4** 5	R^2^ = 0.0828		F(9,7) = 3.39, p = 0.06			R^2^ = 0.0747		R^2^ = 0.1091		R^2^ = 0.0678	
Total retinol+retinyl esters, per 0.64 µ*mol/L*	−0.06±0.04	0.17	0.89	(0.67;1.19)	0.41	−0.00±0.02	0.97	+0.03±0.02	0.18	**+0.04±0.01**	**0.011**
Total carotenoids, per 0.82 µ*mol/L*	−0.04±0.04	0.32	0.78	(0.51;1.20)	0.25	−0.02±0.02	0.39	**+0.07±0.03**	**0.029**	**−0.04±0.02**	**0.044**
Vitamin E, per 12.2 µ*mol/L*	+0.01±0.04	0.81	0.92	(0.69;1.23)	0.56	−0.03±0.02	0.15	−0.04±0.02	0.10	+0.02±0.02	0.39
Vitamin C, per 26.6 µ*mol/L*	+0.03±0.05	0.56	0.87	(0.69;1.11)	0.24	+0.01±0.03	0.64	−0.03±0.03	0.31	**−0.03±0.01**	**0.043**
Folate, per 20.3 *nmol/L*	+0.04±0.03	0.12	0.96	(0.71;1.29)	0.75	−0.03±0.01	0.05	−0.02±0.02	0.27	**−0.06±0.02**	**0.005**
Vitamin B-12, per 1154.4 *pmol/L*	**−0.08±0.03**	**0.006**	1.08	(0.57;2.07)	0.80	−0.00±0.01	0.71	**+0.02±0.01**	**0.024**	**+0.03±0.01**	**0.042**
tHcy, per 4.9 µ*mol/L*	**−0.08±0.03**	**0.015**	0.80	(0.47;1.37)	0.40	−0.03±0.03	0.28	+0.01±0.02	0.58	+0.01±0.03	0.80
25(OH)D, per 8.7 *ng/mL*	+0.07±0.04	0.11	**0.77**	**(0.61;0.96)**	**0.024**	+0.01±0.02	0.55	**−0.08±0.02**	**0.005**	−0.01±0.02	0.57

*Abbreviations*: BMI = Body Mass Index; CI = confidence interval; Met = Metabolic Equivalent; n-3 HUFA = omega-3 highly unsaturated fatty acids; NHANES = National Health and Nutrition Examination Survey; OR = odds ratio; SEE = Standard error of the estimate.

1 Values are odds ratios with 95% confidence intervals or β±SEE. Sampling design complexity is taken into account in all analyses.

2 Model 1 included each nutritional biomarker exposure separately and adjusted for socio-demographic factors: age, sex, race/ethnicity, marital status, educational level and poverty income ratio, and other potential confounders: Lifestyle and health-related factors (smoking status, BMI, physical activity: Mets.hr.wk^−1^, history of selected chronic conditions (i.e. type 2 diabetes, CVD and cancer)), anti-depressant use and dietary intakes (total energy intake, alcohol, caffeine, fiber, *n*-3 HUFA, each of the five carotenoids, vitamin C, vitamin E, folate, vitamin B-12), fruit and vegetable intake, supplement use, anti-depressant use, and the inverse mills ratio, 2-stage Heckman selection model.

3 Model 2 included all serum nutritional biomarker exposures simultaneously, controlling for the same covariate as above.

4 Model 3 is model 1 (i.e. controlling for the same covariates as above) but with main exposures being total retinol+retinyl esters and total carotenoids. The other nutritional biomarkers are not shown because their results were already presented in model 1.

5 Model 4 is model 2 (i.e. controlling for the same covariates as above) but with main exposures being total retinol+retinyl esters, total carotenoids and the other serum nutritional biomarkers entered into the model simultaneously.

In **model 2**, when all serum nutritional biomarkers were entered into the same model simultaneously along with potential confounding covariates, some of the previously observed inverse associations remained significant (though slightly attenuated), but not others. In particular, after correction for multiple testing, while the association between 25(OH)D and sleep disorders became non-significant, the inverse relationship between 25(OH)D and poor sleep quality was retained with respect to Factor 2 (Sleepiness) (p = 0.005). A consistent association was noted for vitamin B-12 with sleep duration and for folate with Factor 3 (Sleep disturbance). However, other associations emerged due to covarying all those nutritional biomarkers together in the same model, particularly individual carotenoids. Most notably, lutein+zeaxanthin were associated with Factor 1, reflecting a deleterious effect on poor sleep-related daytime dysfunction (for each 1 SD = 0.17; β = +0.04±0.01, p = 0.005). Moreover, while *α*-carotene showed an independent inverse association with Factor 2 (Sleepiness), a positive association was found between *β*-carotene and this factor.

In **model 3**, all nutritional biomarkers were entered separately (as in **model 1**) while combining total carotenoids and retinol+retinyl esters together as two main exposures and adjusting for the same covariates. None of the associations for combined total carotenoids and retinol+retinyl esters with the five sleep measures were significant after correcting for multiple testing.

In **model 4**, all nutritional biomarkers were entered simultaneously (as in **model 2**), while combining total carotenoids and retinol+retinyl esters together as two main exposures and adjusting for the same covariates. In this model, significant inverse relationships (p<0.010) were consistently found between vitamin B-12 and sleep duration, 25(OH)D and Factor 2 (Sleepiness), folate and Factor 3 (Sleep disturbance). These associations were not altered when serum total cholesterol was included among potentially confounding factors in **model 4**.

### Sleep disorders and their association with serum nutritional biomarkers

**Figure 1:**
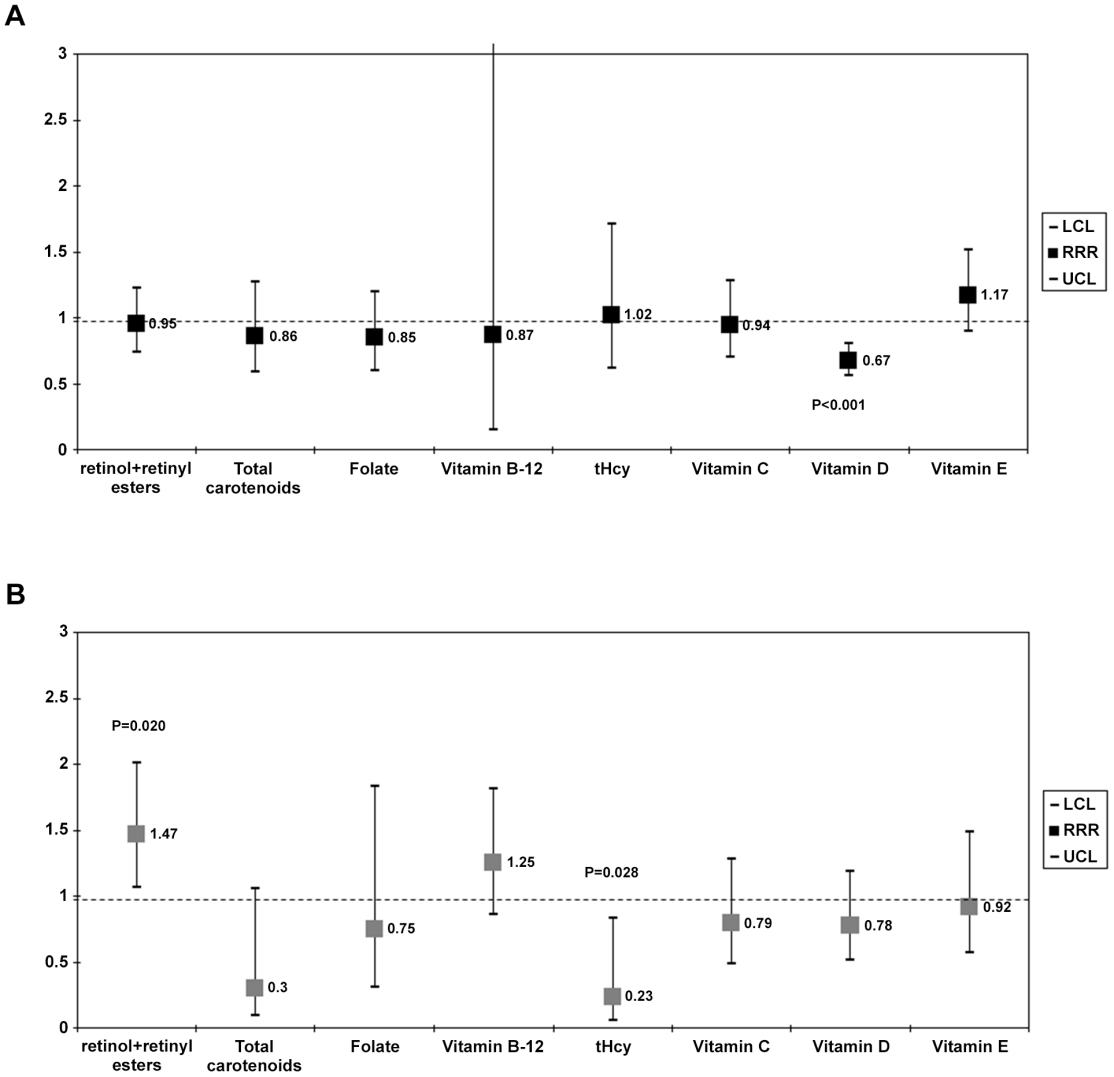
Relative risk ratio (RRR with 95% CI) per 1 SD nutritional biomarker: (A) Insomnia vs. Normal sleep duration^a,b^, (B) Other sleep disorder categories vs. None^a,b^. *Abbreviations*: BMI = Body Mass Index; CI = confidence interval; Met = Metabolic Equivalent; n-3 HUFA = omega-3 highly unsaturated fatty acids; NHANES = National Health and Nutrition Examination Survey; RRR = relative risk ratio; SEE = Standard error of the estimate. ^a^ Values are Relative Risk Ratios (RRR) with 95% confidence intervals. Sampling design complexity is taken into account in all analyses. ^b^ Models included all serum nutritional biomarker exposures simultaneously, and adjusted for socio-demographic factors: age, sex, race/ethnicity, marital status, educational level and poverty income ratio, and other potential confounders: Lifestyle and health-related factors (smoking status, BMI, physical activity: Mets.hr.wk^−1^, history of selected chronic conditions (i.e. type 2 diabetes, CVD and cancer)), anti-depressant use and dietary intakes (total energy intake, alcohol, caffeine, fiber, *n*-3 HUFA, each of the five carotenoids, vitamin C, vitamin E, folate, vitamin B-12), fruit and vegetable intake, supplement use, anti-depressant use, and the inverse mills ratio, 2-stage Heckman selection model as well as serum cholesterol (See **Table 4** **, Model 4** for more details).

Using the same approach as for **model 4** (**Table 4**) and a multiple multinomial logit regression models with outcome being sleep disorder categorized as (0 = none, 1 = insomnia only, 2 = all others), we found that with each 1 SD higher serum level of 25(OH)D there was a 33% lower odds of insomnia (Relative Risk Ratio, RRR = 0.67, 95% CI:0.56–0.80, p<0.001) (**Figures 1A–1B**). All other findings were either null or did not survive familywise bonferroni correction.

### Sleep durations and their association with serum nutritional biomarkers

**Figure 2:**
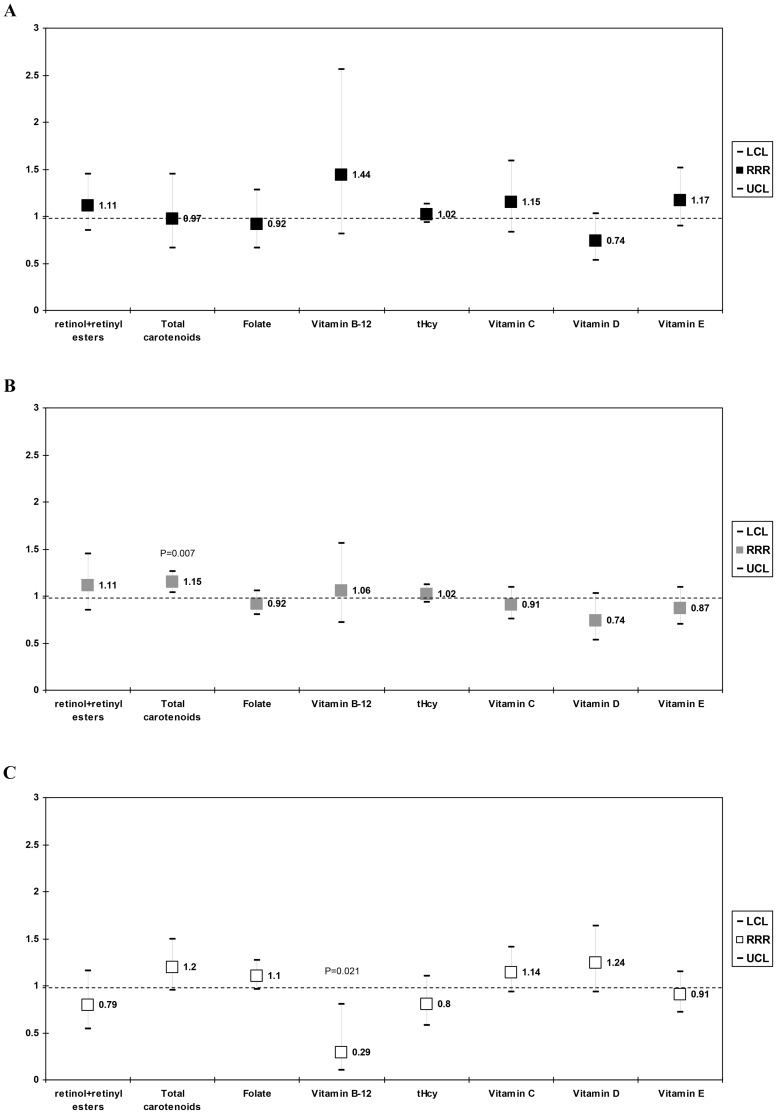
Relative risk ratio (RRR with 95% CI) per 1 SD nutritional biomarker: (A) Very short sleep vs. Normal sleep duration^a,b^, (B) Short sleep vs. Normal sleep duration^a,b^, (C) Long sleep vs. Normal sleep duration^a,b^. *Abbreviations*: BMI = Body Mass Index; CI = confidence interval; Met = Metabolic Equivalent; n-3 HUFA = omega-3 highly unsaturated fatty acids; NHANES = National Health and Nutrition Examination Survey; RRR = relative risk ratio; SEE = Standard error of the estimate. ^a^ Values are Relative Risk Ratios (RRR) with 95% confidence intervals. Sampling design complexity is taken into account in all analyses. ^b^ Models included all serum nutritional biomarker exposures simultaneously, and adjusted for socio-demographic factors: age, sex, race/ethnicity, marital status, educational level and poverty income ratio, and other potential confounders: Lifestyle and health-related factors (smoking status, BMI, physical activity: Mets.hr.wk^−1^, history of selected chronic conditions (i.e. type 2 diabetes, CVD and cancer)), anti-depressant use and dietary intakes (total energy intake, alcohol, caffeine, fiber, *n*-3 HUFA, each of the five carotenoids, vitamin C, vitamin E, folate, vitamin B-12), fruit and vegetable intake, supplement use, anti-depressant use, and the inverse mills ratio, 2-stage Heckman selection model as well as serum cholesterol (See **Table 4** **, Model 4** for more details).

Another multinomial logit model (**Figures 2A–2C**) was conducted with very short, short and long sleep durations being compared to normal sleep duration in their association with serum nutritional biomarker levels, using a similar approach as in **Model 4** (**Table 4**). When the sub-group with very short sleep duration (<5 h per night) was contrasted with normal sleep duration (i.e. 7–8 h per night), only a marginal negative association was found with 25(OH)D and very short sleep (per SD: RRR = 0.74, 95%CI:0.53–1.03, p = 0.07), which did not survive multiple testing correction, (**Figure 2A**). In **Figure 2B**, short sleep (5–6 h per night) was contrasted to normal sleep duration (7–8 h per night). In this case, total carotenoid concentration was linked to increased risk of short sleep (RRR = 1.15, 95%CI:1.04–1.26, p = 0.007), which remained significant after multiple testing correction. Finally, when examining long sleep duration ≥(9 h per night) vs. normal, we found a negative association between vitamin B-12 and extended sleep durations (RRR = 0.29, 95% CI:0.10–0.80, p = 0.021), an association that did not survive multiple testing correction (**Figure 2C**).

## Discussion

To our knowledge, this is the first nationally representative study to examine the association between serum nutritional biomarkers and sleep (quantity and quality) among US adults. Among key findings, independent inverse associations were found between serum vitamin B-12 and sleep duration, 25(OH)D and sleepiness (as well as insomnia), and between folate and sleep disturbance. Serum total carotenoid concentration was linked to higher odds of short sleep duration (i.e. 5–6 h per night) compared to normal sleep duration (7–8 h per night).

### Nutritional biomarkers, diet and sleep: previous studies

Diet and nutritional biomarkers' relationship with sleep quantity/quality and sleep disorders was examined in few previous studies, most of which useddietary intakes as the main exposure.(e.g. [44], [45], [46], [47], [48], [49], [50], [51]) For instance, a recent study of 87 adults (21–45 y), found that insomniacs consumed significantly less energy, carbohydrates, folic acid and vitamin B-12 than normal sleepers [48]. Another study reported that vitamin B-12 intake had a positive psychotropic alerting effect and a distribution of the sleep-wake cycle toward sleep reduction [19]. This specific finding was similar in our study, given that we found serum vitamin B-12 to be associated with shorter sleep duration and a lower propensity for longer sleep duration.

A recent NHANES 2007–08 study observed that sleep duration was associated with dietary intake of various macro and micronutrients. Specifically, short sleep duration was correlated with greater/reduced theobromine, greater/reduced Vitamin C, greater/reduced tap water consumption, greater/reduced lutein+zeaxanthin, greater/reduced lycopene, greater/reduced carbohydrate, greater/reduced selenium. In contrast, long sleep durations were correlated with greater/reduced dodecanoic acid, greater/reduced choline, and greater/reduced alcohol consumption [44].

Another similar NHANES 2007–08 study found that doubling dietary nutrient amount was related to odds of some sleep-related symptoms, but not others, as follows: **(1)** Difficulty falling asleep: α-carotene (OR = 0.96), selenium (OR = 0.80), dodecanoic acid (OR = 0.91), calcium (OR = 0.83) and hexadecanoic acid (OR = 1.10); **(2)** Difficulty maintaining sleep: salt (OR = 1.19), butanoic acid (0.81), carbohydrate (OR = 0.71), dodecanoic acid (OR = 0.90), vitamin D (OR = 0.84), lycopene (OR = 0.98), hexanoic acid (OR = 1.25) and moisture (OR = 1.27); **(3)** Non-restorative sleep: butanoic acid (OR = 1.09), calcium (OR = 0.81), vitamin C (OR = 0.92), water (OR = 0.98), moisture (OR = 1.41) and cholesterol (OR = 1.10); **(4)** Sleepiness: moisture (OR = 1.20), theobromine (OR = 1.04), potassium (OR = 0.70) and water (OR = 0.97) [52]. Although those two studies examined a large number of dietary nutrient intakes rather than a smaller number of serum nutritional biomarkers, the latter found a putative protective effect of vitamin D on certain aspects of sleep quality [52]. In addition, our study found that lutein+zeaxanthin was associated with poorer daytime dysfunction independently of other carotenoid and biomarker levels. This finding is similar to the observed shortened sleep duration associated with intake of lutein+zeaxanthin in one of the two recent studies using NHANES 2007–08 [44]. In contrast to the previous study [52], however, the current study did not observe a significant association between sleep latency and α-carotene intake, but did observe an inverse relationship between α-carotene and sleepiness.

Many studies with serum nutritional biomarkers as predictors of sleep have commonalities with the present study's results, with a few showing an opposite trend. In terms of the relationship between serum 25(OH)D and sleep, low levels in one study was correlated with excessive daytime sleepiness among blacks but not among whites in a consecutive sample of 81 sleep clinic patients [18]. This finding was highly comparable to ours. Given that we had a larger sample size, we found this result in the entire multi-ethnic NHANES sample. In addition, we found an association between 25(OH)D and lower risk of sleep disorders which did not survive correction for multiple testing (0.01≤p<0.05), though was significant in the case of insomnia. In contrast, this association was found specifically for obstructive sleep apnea and restless leg syndrome in two previous studies [21], [22]. In contrast to our findings, Day and colleagues [20] found that patients with obstructive lung apnea, as compared to controls, had lower serum levels of retinyl palmitate and 9-cis retinoic acid, as well as lower levels of all-trans-β-carotene and 9-cis-β-carotene. Moreover, low serum folate has been associated with restless leg syndrome among pregnant women [23]. Higher tHcy was linked to an increased risk of obstructive sleep apnea in some studies [24], [25] but not others [79], [80]. Despite evidence of a protective effect of vitamins C and E on sleep quality [26], [44], [81], our study did not find an independent association between these two antioxidants and the sleep measures under study.

### Potential biological mechanisms of the association of B-vitamins, vitamin D and pro-vitamin A carotenoids with sleep

The sleep-wake cycle is controlled by a master clock in the suprachiasmatic nuclei of the hypothalamus and is reset daily by retina-perceived light [82]. Folate and vitamin B-12 allow remethylation of homocysteine to methionine, thereby improving histone and DNA methylation patterns [83]. These patterns are associated with daily endogenous circadian rhythmicity of the clock protein PER2 [84] and the neuropeptide vasopressin, in the suprachiasmatic nuclei [85]. As a result, these effects entrain the endogenous circadian component of sleep propensity [86] and increase light sensitivity and photic synchronization by affecting melatonin metabolism [87]. However, as stated earlier, a positive psychotropic alerting effect of vitamin B-12 was found in a previous study, possibly due to a distribution of the sleep-wake cycle toward sleep reduction [19]. Moreover, this study suggested that increasing levels of vitamin B-12 may lead to faster decline in melatonin levels, thus reducing the odds of longer sleep duration.

Vitamin D receptors are abundantly expressed in the anterior and posterior hypothalamus, substantia nigra, midbrain central gray, raphe nuclei, and the nucleus reticularis pontis oralis and caudalis, which appear to coordinate the sleep-wake state and the paralysis of the bulbar and somatic musculature during sleep [88]. There may be a narrow range of 25(OH)vitamin D3 blood levels that are necessary to produce normal sleep and clinical trials are needed to determine the effects of Vitamin D3 replacement in the prevention of sleep disorders such as insomnia, obstructive sleep apnea and rapid eye movement related apnea [89].

The importance of Pro-Vitamin A carotenoids and its derivatives on gene transcription in the CNS are presumed to be mediated largely by retinoic acid and its isomer 9-cis-RA interactions with its nuclear receptors RAR and RXR [90]. Recently, it was reported that RARβ (an RAR subtype) gene expression in the hypothalamus determines delta oscillation's contribution in mouse sleep electroencephalogram (EEG) patterns [91]. A 4-week dietary deficiency of vitamin A (VAD) causes the attenuation of delta power in sleep and spontaneous activity in mice. Similarly, chronic administration of LE540, an antagonist of RARs, attenuated wakefulness and EEG delta power during non-rapid eye movement sleep leading to hypo-expression of dopamine D1 receptors and a shortened sleep-wake cycle [92]. In our study, serum total carotenoid concentration was linked to higher odds of short sleep duration (i.e. 5–6 h per night) compared to normal sleep duration (7–8 h per night).

### Strengths and limitations

Our study has numerous strengths. These include using a large nationally representative sample which collected data on a wide range of nutritional biomarkers, and the use of a comprehensive screening instrument to obtain several measures of sleep quality, rather than focusing solely on sleep duration and/or sleep disorders, as was done in previous studies. Limitations of our study, include using a crude measure for dietary supplements and self-reported sleep measures, the study's cross-sectional design which precluded causal inference through ascertaining directionality of relationships, the self-reported sleep measures, the relatively high proportion of missing data from original eligible adult sample resulting in potential selection bias, and residual confounding by unmeasured covariates.

### Conclusion

In sum, a few of the selected serum nutritional biomarkers were associated with sleep quantity and quality. In particular, there was a consistent association indicative of a potential protective effect of serum 25(OH)D and folate on two dimensions of sleep quality, namely sleepiness and sleep disturbance. In contrast, total carotenoids were positively associated with shorter sleep duration and a similar deleterious effect of serum vitamin B-12 on sleep duration was found. This highlights the specificity of each of those micronutrients in their relationship with sleep. However, longitudinal studies are needed in which baseline nutritional biomarkers are modeled against change in sleep measures to ascertain temporality and assess putative causal relationships.

## Supporting Information

File S1 **Sleep quality-related measures; factor analysis approach and results; Correlation matrix between biomarkers.** Method S1, Sleep quality-related measures and factor analytic approach. Table S1, Rotated three-factor model solution: All 21 items. Table S2, Rotated three-factor model solution: reduced to 15 items. Table S3, Confirmatory factor analysis with 15 items, related fit indices, and correlation between EFA and CFA predicted factors. Table S4, Correlation matrix between key serum nutritional biomarkers.
(DOC)
