# Supplementary material for: Serum Nutritional Biomarkers and Their Associations with Sleep among US Adults in Recent National Surveys
Source: PLoS One. 2014 Aug 19;9(8):e103490. doi: 10.1371/journal.pone.0103490 (PMC4138077; doi:10.1371/journal.pone.0103490)
Supplement: File S1 — Sleep quality-related measures; factor analysis approach and results; Correlation matrix between biomarkers. Method S1, Sleep quality-related measures and factor analytic approach. Table S1, Rotated three-factor model solution: All 21 items. Table S2, Rotated three-factor model solution: reduced to 15 items. Table S3, Confirmatory factor analysis with 15 items, related fit indices, and correlation between EFA and CFA predicted factors. Table S4, Correlation matrix between key serum nutritional biomarkers. (DOC) [file pone.0103490.s001.doc]

**FILE S1: Sleep quality-related measures; factor analysis approach and resutls; Correlation matrix between biomarkers**

**Method S1: Sleep quality-related measures and Factor analytic approach**

The following questions were used to obtain the first two sleep quality related measures:

**(A) Sleep duration**:

How much sleep do you get (hours)? The range is 1-11 and is truncated at 12 or more. Response of “I don’t know” or “refused” were considered missing: **sld010h**

**(B) Sleep disorders**:

Ever told by doctor that you have a sleep disorder? Yes/No: **slq060**

If yes, which one?

Sleep apnea, Yes/No: **slq070A**

Insomnia, Yes/No: **slq070B**

Restless leg syndrome, Yes/No: **slq070C**

Other, Yes/No: **slq070D**

The participant should have answered yes on at least one of the sleep disorders to be considered as having a sleep disorder. If he/she answered yes on the general question but had a missing value on the type, they were considered missing.

**(C) Factor analysis of the sleep quality-related measures (only ordinal measures on likert-scale):**

The following measures were included at first into the factor analysis (code 77=Refused, 99=don’t know considered as missing)

(**C.1.**) How much sleep do you get (hours), (same as **A**), NHANES variable name: **sld010h**

(**C.2.**) How long to fall asleep (min.), 0-50, 60 or more=60: **sld020m**

(**C.3.**) How often do you snore?, 4-point likert scale: 0=never, 1=Rarely (1-2 nights/week), 2= Occasionally (3-4 nights/week), 3=Frequently (5 or more nights/week): **slq030**

(**C.4.**) How often do you snort/stop breathing? 4-point likert scale: 0=never, 1=Rarely (1-2 nights/week), 2= Occasionally (3-4 nights/week), 3=Frequently (5 or more nights/week): **slq040**

**(C.5)** How often do you have trouble falling asleep? 5-point likert scale: 0=Never, 1=Rarely (1 time a month), 2=Sometimes (2-4 times a month), 3=Often (5-15 times a month), 4=Almost always (16-30 times a month) : **slq080**

**(C.6.)** How often do you wake up during the night? 5-point likert scale: 0=Never, 1=Rarely (1 time a month), 2=Sometimes (2-4 times a month), 3=Often (5-15 times a month), 4=Almost always (16-30 times a month) : **slq090**

**(C.7.)** How often do you wake up too early in the morning? 5-point likert scale: 0=Never, 1=Rarely (1 time a month), 2=Sometimes (2-4 times a month), 3=Often (5-15 times a month), 4=Almost always (16-30 times a month) : **slq100**

**(C.8.)** How often do you feel unrested during the day? 5-point likert scale: 0=Never, 1=Rarely (1 time a month), 2=Sometimes (2-4 times a month), 3=Often (5-15 times a month), 4=Almost always (16-30 times a month) : **slq110**

**(C.9.)** How often do you feel overly sleepy during the day? 5-point likert scale: 0=Never, 1=Rarely (1 time a month), 2=Sometimes (2-4 times a month), 3=Often (5-15 times a month), 4=Almost always (16-30 times a month) : **slq120**

**(C.10.)** How often did you not get enough sleep? 5-point likert scale: 0=Never, 1=Rarely (1 time a month), 2=Sometimes (2-4 times a month), 3=Often (5-15 times a month), 4=Almost always (16-30 times a month) : **slq130**

**(C.11.)** How often do you take pills to help you sleep? 5-point likert scale: 0=Never, 1=Rarely (1 time a month), 2=Sometimes (2-4 times a month), 3=Often (5-15 times a month), 4=Almost always (16-30 times a month) : **slq140**

**(C.12.)** How often do you have leg jerks while sleeping? 5-point likert scale: 0=Never, 1=Rarely (1 time a month), 2=Sometimes (2-4 times a month), 3=Often (5-15 times a month), 4=Almost always (16-30 times a month) : **slq150**

**(C.13.)** How often do you have leg cramps while sleeping? 5-point likert scale: 0=Never, 1=Rarely (1 time a month), 2=Sometimes (2-4 times a month), 3=Often (5-15 times a month), 4=Almost always (16-30 times a month) : **slq160**

**(C.14.)** Do you have difficulty concentrating when tired? 5-point likert scale: 1=Don’t do this activity for other reasons, 2=no difficulty, 3=Yes, a little difficulty, 4=Yes, moderate difficulty, 5=yes, extreme difficulty: **slq170**

**(C.15.)** Do you have difficulty remembering when tired? 5-point likert scale: 1=Don’t do this activity for other reasons, 2=no difficulty, 3=Yes, a little difficulty, 4=Yes, moderate difficulty, 5=yes, extreme difficulty: **slq180**

**(C.16)** Do you have difficulty eating when tired? 5-point likert scale: 1=Don’t do this activity for other reasons, 2=no difficulty, 3=Yes, a little difficulty, 4=Yes, moderate difficulty, 5=yes, extreme difficulty: **slq190**

**(C.17.)** Do you have difficulty with a hobby when tired? 5-point likert scale: 1=Don’t do this activity for other reasons, 2=no difficulty, 3=Yes, a little difficulty, 4=Yes, moderate difficulty, 5=yes, extreme difficulty: **slq200**

**(C.18.)** Do you have difficulty getting things done when tired? 5-point likert scale: 1=Don’t do this activity for other reasons, 2=no difficulty, 3=Yes, a little difficulty, 4=Yes, moderate difficulty, 5=yes, extreme difficulty: **slq210**

**(C.19.)** Do you have difficulty with finances when tired? 5-point likert scale: 1=Don’t do this activity for other reasons, 2=no difficulty, 3=Yes, a little difficulty, 4=Yes, moderate difficulty, 5=yes, extreme difficulty: **slq220**

**(C.20.)** Do you have difficulty with work when tired? 5-point likert scale: 1=Don’t do this activity for other reasons, 2=no difficulty, 3=Yes, a little difficulty, 4=Yes, moderate difficulty, 5=yes, extreme difficulty: **slq230**

**(C.21.)** Do you have difficulty using the phone when tired? 5-point likert scale: 1=Don’t do this activity for other reasons, 2=no difficulty, 3=Yes, a little difficulty, 4=Yes, moderate difficulty, 5=yes, extreme difficulty: **slq240**

At a first stage of the factor analytic procedure, 15 items out of the 23 were found to provide a simple structure for a 3-factor model, so those were selected for another 3-factor model. Those items were **C.2**., **C.5** to **C.7 (Factor 3), C.8 to C.10 (Factor 2),**  and **C.14** to **C.21 (Factor 1)**. The results of the first and second rotated 3-factor model solutions are presented below.

For the 21 items that were measured on a Likert scale of 1-5, a factor analytic approach was used. Consequently, a number of common factors were extracted based on common variance, and factor loadings were estimated with the residual variance being labeled as uniqueness for each of the 21 items. The common factor model can be summarized as follows:

Item_i_= $\sum_{j=1}^{k} \lambda_{ij}*Factor_{j}+\phi_{i}$

Where Item_i_ is the value of the item on a scale of 1-5, λ_ij_ is the factor loading for each item and each factor, Factor_j_ is a standardized z-score for each factor j, and φ_i_ is the residual error, the squared value of which is the uniqueness. The sum of squared factor loadings for each Item_i_ is the communality or the common variance that is accounted for by the extracted factors. An eigenvalue>1 rule was used and the Scree plot was examined to determine the number of extracted factors that would produce the best model fit. The factor loadings were then rotated using varimax orthogonal rotation. Factor loadings of ≥0.40 were considered significant. The factor scores (*z*-scores) were predicted using the regression method and used as markers of a specific domain of sleep quality, with higher scores indicating poorer sleep. Based on those criteria, three factors were extracted. Then, items with weak factor loading on all factors were deleted, yielding 15 items. , Finally, a factor analysis of these 15 items was conducted. Three factors were extracted and rotated using varimax orthogonal rotation: **Factor 1**: “Poor sleep-related daytime dysfunction”; **Factor 2**: “Sleepiness” and **Factor 3**: “Sleep disturbance”. Below were the findings of the exploratory factor analytic approach (**Supplemental Tables 1 and 2**) and the confirmatory factor analysis (CFA, **Supplemental Table 3**). Fit indices for the CFA indicated a reasonable fit (RMSEA, TLI) to close fit (CFI, SRMR and CD). When predicting **Factors 1-3** from the CFA, each correlated highly with its counterpart from the exploratory factor analysis results.

**Table S1. Rotated three-factor model solution: All 21** items

|  | **Factor 1** | **Factor 2** | **Factor 3** | **Uniqueness** |
| --- | --- | --- | --- | --- |
| **C.1** | -0.07 | -0.26 | -0.22 | 0.88 |
| **C.2** | +0.06 | **+0.53** | +0.02 | 0.71 |
| **C.3** | +0.00 | -0.09 | +0.25 | 0.93 |
| **C.4** | +0.03 | -0.07 | +0.30 | 0.90 |
| **C.5** | +0.14 | **+0.71** | +0.19 | 0.44 |
| **C.6** | +0.10 | **+0.69** | +0.29 | 0.42 |
| **C.7** | -0.09 | **+0.58** | +0.29 | 0.57 |
| **C.8** | +0.21 | +0.32 | **+0.66** | 0.42 |
| **C.9** | +0.27 | +0.28 | **+0.65** | 0.42 |
| **C.10** | +0.19 | +0.38 | **+0.57** | 0.49 |
| **C.11** | +0.05 | +0.28 | +0.16 | 0.90 |
| **C.12** | +0.15 | 0.16 | +0.29 | 0.86 |
| **C.13** | +0.12 | +0.18 | +0.27 | 0.88 |
| **C.14** | **+0.62** | +0.13 | +0.33 | 0.49 |
| **C.15** | **+0.59** | +0.14 | +0.29 | 0.55 |
| **C.16** | **+0.42** | +0.09 | -0.00 | 0.82 |
| **C.17** | **+0.52** | +0.11 | +0.17 | 0.68 |
| **C.18** | **+0.66** | +0.09 | 0.15 | 0.53 |
| **C.19** | **+0.64** | +0.10 | 0.11 | 0.56 |
| **C.20** | **+0.56** | +0.03 | +0.05 | 0.69 |
| **C.21** | **+0.56** | +0.11 | +0.07 | 0.67 |

**Table S2. Rotated three-factor model solution: reduced to 15** items

|  | **Factor 1** | **Factor 2** | **Factor 3** | **Uniqueness** |
| --- | --- | --- | --- | --- |
| **C.2** | +0.05 | +0.08 | **+0.51** | 0.73 |
| **C.5** | +0.13 | +0.25 | **+0.69** | 0.45 |
| **C.6** | +0.08 | +0.31 | **+0.70** | 0.41 |
| **C7** | +0.08 | +0.30 | **+0.59** | 0.56 |
| **C.8** | +0.15 | **+0.75** | +0.24 | 0.35 |
| **C.9** | +0.21 | **+0.75** | +0.20 | 0.36 |
| **C.10** | +0.15 | **+0.62** | +0.33 | 0.49 |
| **C.14** | **+0.59** | +0.39 | +0.10 | 0.49 |
| **C.15** | **+0.57** | +0.33 | +0.12 | 0.55 |
| **C.16** | **+0.42** | +0.02 | +0.10 | 0.81 |
| **C.17** | **+0.51** | +0.22 | +0.10 | 0.68 |
| **C.18** | **+0.66** | +0.17 | +0.10 | 0.53 |
| **C.19** | **+0.64** | +0.15 | +0.10 | 0.56 |
| **C.20** | **+0.55** | +0.09 | 0.04 | 0.69 |
| **C.21** | **+0.55** | 0.11 | 0.10 | 0.67 |

**Table S3. Confirmatory factor analysis with 15 items, related fit indices, and correlation between EFA and CFA predicted factors**

|  | **Factor 1** | **Factor 2** | **Factor 3** |
| --- | --- | --- | --- |
| **C.2** | 0 | 0 | **0.44** |
| **C.5** | 0 | 0 | **0.69** |
| **C.6** | 0 | 0 | **0.84** |
| **C7** | 0 | 0 | **0.72** |
| **C.8** | 0 | **0.84** | 0 |
| **C.9** | 0 | **0.82** | 0 |
| **C.10** | 0 | **0.72** | 0 |
| **C.14** | **0.72** | 0 | 0 |
| **C.15** | **0.69** | 0 | 0 |
| **C.16** | **0.40** | 0 | 0 |
| **C.17** | **0.57** | 0 | 0 |
| **C.18** | **0.67** | 0 | 0 |
| **C.19** | **0.65** | 0 | 0 |
| **C.20** | **0.53** | 0 | 0 |
| **C.21** | **0.56** | 0 | 0 |

**Abbreviations:** CD=Coefficient of Determination; CFA=Confirmatory Factor Analysis, CFI=Comparative Fit Index; EFA=Exploratory Factor Analysis, RMSEA=Root Mean Square Error of Approximation, SRMR (Square Root Mean Residuals).

**Fit indices:** RMSEA=0.073 (cutoff of 0.080 for reasonable fit), CFI=0.91, TLI=0.89, SRMR=0.045 (cutoff of 0.05 for good fit), CD=0.992.

Correlation between EFA and CFA predicted factors: Factor1 (r=0.93, p<0.001), Factor 2(r=0.95, p<0.001), Factor 3 (r=0.92, p<0.001).

**Table S4**. Correlation matrix between key serum nutritional biomarkers

|  | α-Carotene | β-Carotene | β-Cryptoxanthin | Lutein+zeaxanthin | Lycopene | Retinol | Retinyl esters | Vitamin E | Vitamin C | Folate | Vitamin B-12 | tHcy | 25(OH)D |
| --- | --- | --- | --- | --- | --- | --- | --- | --- | --- | --- | --- | --- | --- |
| α-Carotene, *μmol/L* | 1 |  |  |  |  |  |  |  |  |  |  |  |  |
| β-Carotene, *μmol/L* | +0.80*** | 1 |  |  |  |  |  |  |  |  |  |  |  |
| β-Cryptoxanthin, *μmol/L* | +0.59*** | +0.61*** | 1 |  |  |  |  |  |  |  |  |  |  |
| Lutein+zeaxanthin, *μmol/L* | +0.59*** | +0.58*** | +0.60*** | 1 |  |  |  |  |  |  |  |  |  |
| Lycopene, *μmol/L* | +0.25*** | +0.28*** | +0.33*** | +0.31*** | 1 |  |  |  |  |  |  |  |  |
| Retinol, *μmol/L* | +0.09*** | +0.08*** | +0.05*** | +0.12*** | +0.31*** | 1 |  |  |  |  |  |  |  |
| Retinyl esters, *μmol/L* | +0.40*** | +0.43*** | +0.34*** | +0.39*** | +0.43*** | +0.24*** | 1 |  |  |  |  |  |  |
| Vitamin E, *μmol/L* | +0.32*** | +0.39*** | +0.28*** | +0.41*** | +0.21*** | +0.41*** | +0.47*** | 1 |  |  |  |  |  |
| Vitamin C, *μmol/L* | +0.41*** | +0.45*** | +0.45*** | +0.35*** | +0.08*** | +0.13*** | +0.13*** | +0.29*** | 1 |  |  |  |  |
| Folate, *nmol/L* | +0.30*** | +0.38*** | +0.22*** | +0.20*** | +0.01 | +0.22*** | +0.31*** | +0.42*** | +0.50*** | 1 |  |  |  |
| Vitamin B-12, *pmol/L* | +0.17*** | +0.24*** | +0.16*** | +0.12*** | -0.00 | +0.05*** | +0.17*** | +0.21*** | +0.21*** | +0.33*** | 1 |  |  |
| tHcy, *μmol/L* | -0.16*** | -0.15*** | -0.19*** | -0.08*** | -0.13*** | +0.24*** | -0.11*** | -0.00 | -0.20*** | -0.22*** | -0.25*** | 1 |  |
| 25(OH)D, *ng/mL* | +0.17*** | +0.20*** | +0.07*** | +0.06*** | +0.07*** | +0.27*** | +0.20*** | +0.21*** | +0.21*** | +0.28*** | +0.14*** | -0.12*** | 1 |

***p<0.01 for null hypothesis that r=0.
